# Social determinants of dementia risk in individuals with mild cognitive impairment: a systematic review

**DOI:** 10.1002/alz70860_106358

**Published:** 2025-12-23

**Authors:** Rifah Anjum, Natalia Chemas, Avinash Chandra, Sedigheh Zabihi, Beth Stuart, Claudia Cooper, Charles R Marshall

**Affiliations:** ^1^ Queen Mary University of London, London, Greater London, United Kingdom; ^2^ Queen Mary University of London, London, United Kingdom

## Abstract

**Background:**

Patients with mild cognitive impairment (MCI) are at high risk for dementia. This prodrome is characterised by mild memory problems but no functional impairment. Social determinants of health (SDH) are the non‐medical factors which influence health outcomes and can influence dementia risk in cognitively normal individuals. However, whether SDH can influence progression to dementia over time in individuals with MCI is unclear. This systematic review aimed to review the evidence of the association between SDH, particularly at the level of an individuals’ socioeconomic position, and the likelihood of developing dementia in individuals with MCI.

**Method:**

The PubMed and Web of Science biomedical electronic databases were searched to identify longitudinal cohort studies. Eligible studies included MCI at baseline (or the closely related concept of cognitive impairment without dementia), an outcome of dementia, and a follow‐up period of at least one year. Only studies reporting SDH, particularly at the level of an individual's socioeconomic position based on the WHO Conceptual Framework, were included in this review. This includes the following keywords: socioeconomic position, social class, gender/sex, ethnicity/race, racism, education, occupation and income. Eligible articles were screened independently, and the Newcastle‐Ottawa Scale was used to assess the risk of bias of each study.

**Result:**

A total of 2,082 articles were identified and screened. Approximately 20 studies met the eligibility criteria with education being the most common SDH reported. A small but protective effect of education on the risk of conversion from MCI to dementia was found although the results appear inconsistent. Additionally, preliminary evidence suggests that gender/sex does not influence likelihood of conversion.

**Conclusion:**

SDH may influence the progression from MCI to dementia over time, in particular educational attainment. Based on these results, interventions promoting early‐life obtainment of education may be warranted. However, more evidence is required to clarify the causality of this relationship. More studies are warranted to improve knowledge on the role of SDH in dementia risk in MCI patients who are already at risk of dementia.

